# Pharmacological reduction of neutrophil infiltration reduces *Clostridioides difficile* infection severity

**DOI:** 10.1128/mbio.03430-25

**Published:** 2026-03-30

**Authors:** Orlaith Keenan, Joshua Soto Ocaña, Alexa Semon, Tiffany H. Zhou, Kassy Donohoe, Emma E. Furth, Gavyn Chern Wei Bee, Daniel L. Aldridge, Juliana Diamantino, Christopher A. Hunter, Ken Cadwell, David M. Aronoff, Joseph P. Zackular

**Affiliations:** 1 Department of Pathology and Laboratory Medicine, Perelman School of Medicine, University of Pennsylvania 14640 , Philadelphia, Pennsylvania, USA; 2 Department of Pathology and Laboratory Medicine, Children’s Hospital of Philadelphia, Philadelphia, Pennsylvania, USA; 3 Institute for Immunology and Immune Health, Perelman School of Medicine, University of Pennsylvania 14640 , Philadelphia, Pennsylvania, USA; 4 Division of Gastroenterology and Hepatology, Department of Medicine, University of Pennsylvania Perelman School of Medicine 14640 , Philadelphia, Pennsylvania, USA; 5 Department of Pathobiology, University of Pennsylvania School of Veterinary Medicine 70736 , Philadelphia, Pennsylvania, USA; 6 Division of Infectious Diseases, Department of Medicine, Indiana University School of Medicine 12250, Indianapolis, Indiana, USA; 7 The Center for Microbial Medicine, Children’s Hospital of Philadelphia, Philadelphia, Pennsylvania, USA; Universite de Geneve, Geneva, Switzerland

**Keywords:** *Clostridium difficile*, immune response, neutrophils, host-pathogen interactions

## Abstract

*Clostridioides difficile* is the leading cause of nosocomial infections and an urgent public health threat. This bacterial pathogen is challenging to treat due to antibiotic resistance and high recurrence rates, highlighting the need for additional therapeutic strategies. Host inflammatory responses are major drivers of disease severity during *C. difficile* infection (CDI), yet few therapeutic strategies target these pathways. Here, we show that the prostaglandin E_1_ (PGE_1_) analog misoprostol markedly reduces CDI severity by modulating host immune responses. During CDI, misoprostol decreases circulating neutrophils and limits infiltration into the colon, reducing epithelial damage, intestinal pathology, and infection severity. Additionally, misoprostol reduces serum granulocyte colony-stimulating factor (G-CSF), an important cytokine in neutrophil mobilization, controlling neutrophil levels during CDI. Together, these findings highlight neutrophil infiltration as a key driver of *C. difficile-*associated disease and identify innate immune modulation as a potential host-directed therapeutic strategy.

**IMPORTANCE:**

*Clostridioides difficile* remains a leading cause of antibiotic-associated diarrhea worldwide and a major healthcare burden. While antibiotics are commonly used to treat CDI, high antibiotic resistance and recurrence rates highlight the need for additional therapeutic approaches. Here, we demonstrate that modulation of the innate immune response can mitigate disease severity. Administration of the prostaglandin analog misoprostol reduces neutrophil availability during CDI, resulting in decreased inflammation and improved outcomes. These findings suggest that fine-tuning neutrophil responses may present a promising host-directed strategy to improve CDI outcomes.

## INTRODUCTION

*Clostridioides difficile* is a leading cause of antibiotic-associated diarrhea and remains a major public health threat worldwide (1, 2). *C. difficile* causes a wide range of disease outcomes and is challenging to treat due to high rates of antibiotic resistance, recurrence, and limited therapeutic options. Antibiotic use is the primary risk factor for *C. difficile* infection (CDI), as it disrupts the resident microbiota, allowing *C. difficile* spores to germinate and produce toxins that damage the intestinal epithelium and trigger a strong inflammatory response (3). This inflammation correlates strongly with CDI severity (4, 5), highlighting the central role of the immune response in shaping CDI pathology. Although antibiotics remain the leading treatment, they fail to resolve toxin-induced inflammation, and current immunotherapeutic approaches, including monoclonal antibodies and vaccines, primarily neutralize toxins rather than host inflammatory responses (6, 7). As a result, there remains a need for host-directed approaches that modulate immune responses to improve CDI outcomes.

The innate immune response plays a pivotal role in shaping infection outcomes (4, 5). Following toxin-mediated epithelial damage, neutrophils and monocytes are rapidly recruited to the gut, where they amplify inflammation through cytokine and chemokine production (8). Neutrophil influx into the epithelium and submucosa is a hallmark of CDI and can be both protective and pathogenic (9–11). While neutrophils are important for controlling infection, they also release inflammatory molecules that can damage the epithelium and impair wound healing in colitis (12, 13). Neutrophil depletion in murine models demonstrates that these cells are necessary for host survival during acute infection (9, 11). However, excessive neutrophil recruitment exacerbates pathology during CDI (10, 14) and can contribute to pseudomembrane formation during severe disease (15). Together, these findings highlight the importance of a balanced neutrophil response during CDI, although the mechanisms regulating this balance remain unclear. A more complete understanding of neutrophil biology during CDI could thus yield promising targets for therapeutic intervention.

Prostaglandins (PGs) are short-lived lipid mediators produced through the arachidonic acid pathway that play important roles in maintaining intestinal homeostasis and regulating inflammation (16). We previously reported that misoprostol, an FDA-approved stable PGE_1_ analog that pharmacologically mimics the endogenous molecule PGE_2_ (17), protects mice from CDI by improving survival, reducing intestinal permeability, and promoting recovery of the microbiota following antibiotic perturbation (18). However, the mechanisms underlying this protection remain unclear. Misoprostol binds and activates E prostanoid (EP) receptors, including EP1, EP2, EP3, and EP4, which are expressed on a variety of cells, including intestinal epithelial cells and immune cells, and mediate diverse, context-specific inflammatory and homeostatic responses (16, 17, 19). While misoprostol has known cytoprotective effects in the gastrointestinal tract (20), whether it acts by modulating *C. difficile* virulence, enhancing epithelial barrier integrity, or regulating host immune responses during CDI has not been defined. PGE_2_, the most abundant PG, has well-described roles in limiting intestinal inflammation and exerts context-specific effects on epithelial homeostasis and immune function in the gut (21–24). PGE_2_ promotes epithelial repair and modulates inflammatory responses during colitis and intestinal infection (21, 25–27). While the roles of PGs during CDI remain poorly understood, these findings suggest that PGs may restrict inflammation and promote mucosal repair during CDI.

In this study, we demonstrate that misoprostol mitigates CDI severity by improving intestinal barrier function and modulating host inflammatory responses. We report that misoprostol protects against severe CDI by reducing circulating neutrophils in the blood and limiting their influx into the colon. We further show that misoprostol controls neutrophil levels during CDI by reducing the serum levels of granulocyte colony-stimulating factor (G-CSF), an important cytokine in neutrophil development and mobilization (28). Conversely, G-CSF supplementation attenuates the protective effects of this treatment. These findings provide new insight into the contribution of neutrophils to *C. difficile* pathogenesis and highlight how pharmacologic modulation of the host response can be leveraged to develop effective host-directed therapies for CDI.

## RESULTS

### Misoprostol reduces CDI severity by limiting colonic inflammation and epithelial injury

**Fig 1:**
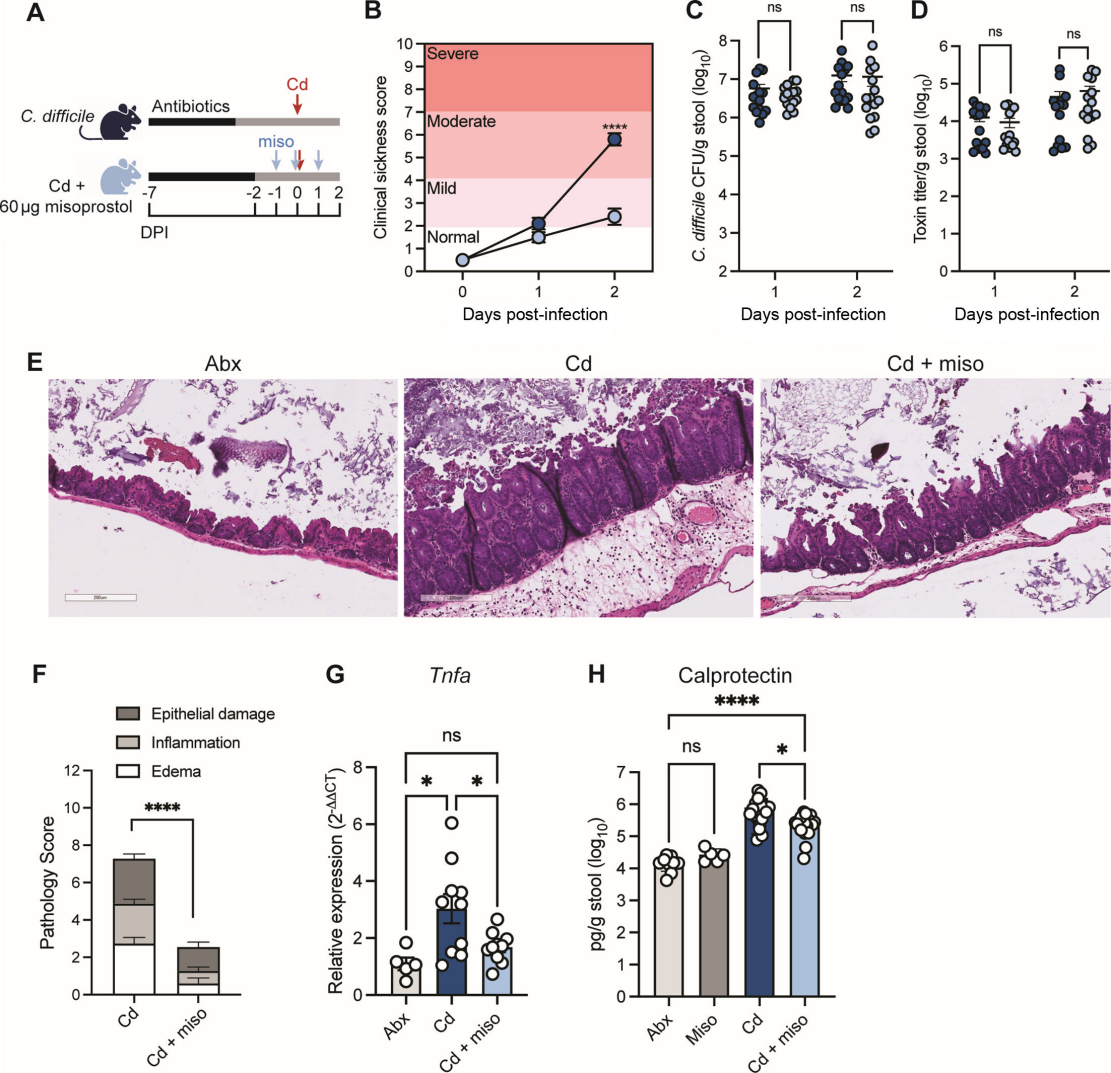
Misoprostol reduces CDI severity by limiting colonic inflammation and epithelial damage. (**A**) Schematic of experimental design. Mice were treated with antibiotics (Abx) in their drinking water and infected with 1 × 10^5^ *C. difficile* spores (Cd). Misoprostol-treated mice (Miso or Cd + miso) were administered 60 μg misoprostol via intraperitoneal (i.p.) injection daily from −1 days post-infection (DPI) to 1 DPI. (**B**) Composite score of weight loss, stool consistency, and behavior (*N* = 15 per group). (**C**) *C. difficile* colony-forming units (CFU) and (**D**) stool toxin titers at 2 DPI, quantified by a cell rounding assay measuring total cytotoxic activity (*N* = 15 per group). (**E**) Representative images of H&E-stained cecal slides from infected mice at 2 DPI. (**F**) Pathology scores from H&E-stained cecal slides at 2 DPI (*N* = 15 per group). (**G**) RT-qPCR on bulk colon tissue harvested from mice at 2 DPI. Data relative to average *Gapdh* expression of uninfected antibiotic-treated mice (Abx) (*N* = 5–10 per group). (**H**) Fecal calprotectin levels measured by ELISA at 2 DPI. (*N* = 5–15 per group). (**B–H**) Data represented as mean ± SEM and representative of at least two independent experiments. Statistics were performed by two-way ANOVA with Sidak’s multiple comparisons (**B**), Mann-Whitney multiple comparison test (**C and D**), Welch’s unpaired *t*-test (**F**), and one-way ANOVA with Tukey’s multiple comparisons (**G**) or Dunnett’s T3 multiple comparisons (**H**). ns, (not significant), *P* > 0.05; *P < 0.05; ****P < 0.0001.

Pharmacologic modulation of host immune responses during CDI remains a promising avenue for treatment of severe acute infection. Our previous work demonstrated that misoprostol has therapeutic potential for CDI, rescuing mice from lethal infection (18). However, the mechanisms underlying this protection remain unclear. To further define mechanisms of misoprostol’s protective effects during acute CDI, we leveraged a mouse model of infection in which misoprostol was administered daily by intraperitoneal (i.p.) injection beginning one day prior to infection with *C. difficile* (Fig. 1A). Consistent with previous findings, misoprostol-treated mice (Cd + miso) exhibited less severe disease during acute CDI compared to untreated *C. difficile*-infected mice (Cd), as measured by a composite score that evaluates weight loss, stool consistency, and moribund behavior (29) (Fig. 1B). Given the clinical importance of disease resolution and recurrence in CDI, we also asked whether misoprostol influences susceptibility to recurrent infection following resolution of acute disease. Despite its robust protective effects during acute CDI, misoprostol treatment did not reduce the incidence of CDI recurrence (Fig. S1), suggesting that misoprostol attenuates acute disease severity without conferring resistance to relapse.

Next, we sought to define the mechanism of misoprostol-mediated protection during acute infection. The impact of misoprostol on pathogen dynamics has not been established, so we first measured *C. difficile* burdens and toxin production. Notably, misoprostol treatment did not affect the abundance of *C. difficile* or stool toxin titers throughout the infection (Fig. 1C and D), suggesting that PG treatment does not impact *C. difficile*’s major virulence factors and instead likely modulates the host response to reduce CDI severity.

Based on these findings, and given the role of PGs as known immunomodulators (16, 23, 24), we postulated that the protective effects of misoprostol were through modulation of the host response to infection. To assess this, we first quantified intestinal pathology of H&E-stained cecal sections and found that misoprostol treatment significantly reduced histological markers of CDI severity (Fig. 1E and F). Consistent with reduced pathology, Cd + miso mice also exhibited reduced canonical markers of inflammation, including reduced colonic expression of the inflammatory cytokine TNF-α to levels observed in uninfected antibiotic-treated controls (Abx) (Fig. 1G). Additionally, levels of fecal calprotectin, a neutrophil-derived protein and marker of intestinal inflammation (30), were significantly reduced in Cd + miso mice compared to Cd mice (Fig. 1H).

**Fig 2:**
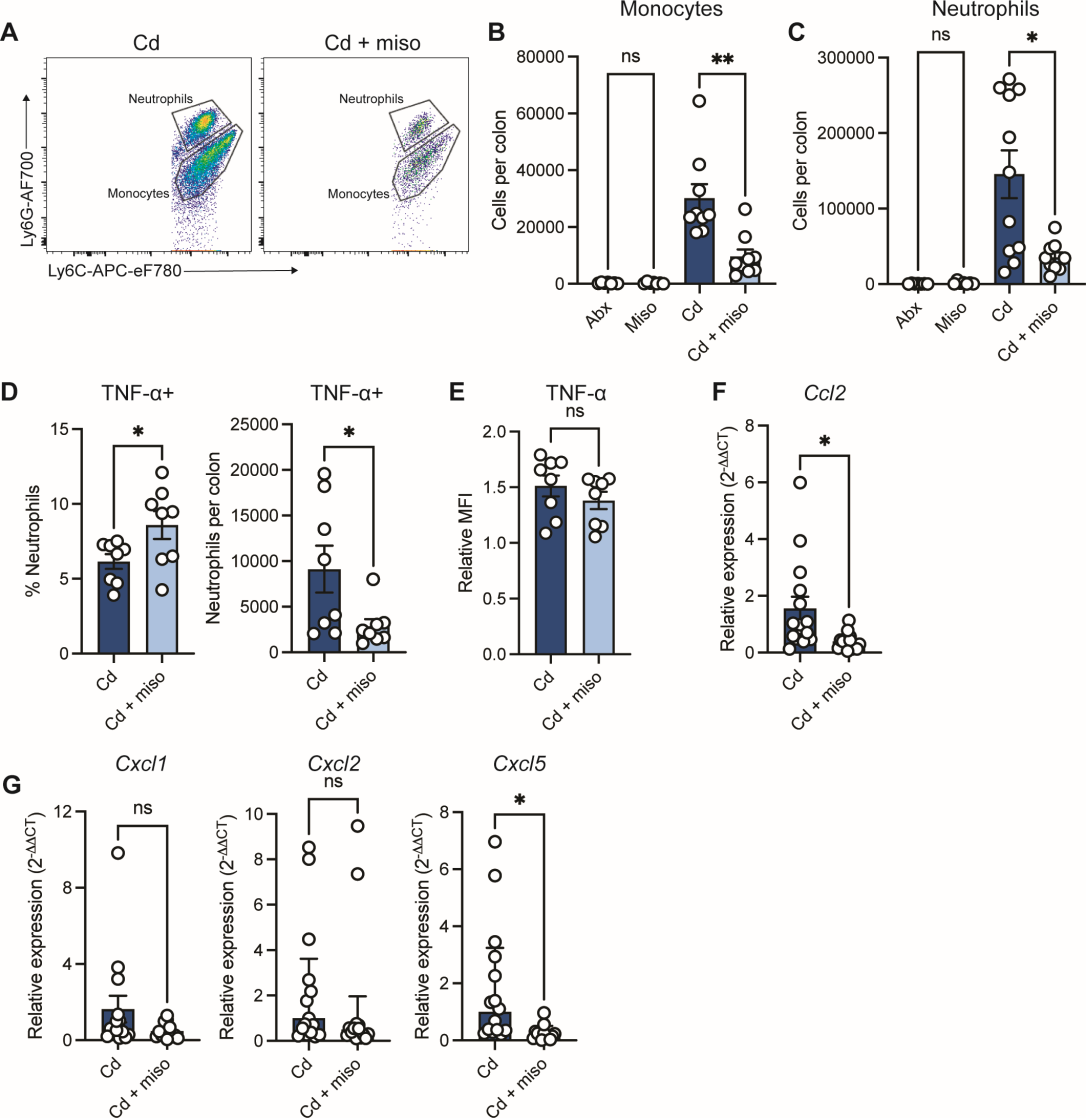
Misoprostol reduces colonic monocyte and neutrophil infiltration during CDI. (**A–G**) Mice were infected with *C. difficile* (Cd) and treated with or without misoprostol (miso). Samples were collected 2 days post-infection. (**A**) Representative flow cytometry plots of monocytes and neutrophils in the cLP. (**B and C**) Quantification of total (**B**) monocytes and (**C**) neutrophils in the cLP. Antibiotic (Abx) and misoprostol (Miso) treated uninfected controls (*N* = 7–9 per group, representative of two independent experiments). (**D and E**) (**D**) Frequency and total number of TNF-α positive neutrophils in the cLP and (**E**) TNF-α MFI of neutrophils relative to antibiotic-treated uninfected controls (*N* = 8 per group, representative of two independent experiments). (**F and G**) RT-qPCR of (**F**) monocyte- and (**G**) neutrophil-recruiting chemokines in bulk colon tissue harvested from mice. Data relative to average *Gapdh* expression of Cd mice (*N* = 15 per group, representative of three independent experiments). (**B–G**) Each dot represents an individual mouse. Data are represented as mean ± SEM. Statistics by one-way ANOVA test with Tukey’s multiple comparisons (**B and C**) and Welch’s *t*-test (**D–G**). ns (not significant), *P* > 0.05; *P < 0.05; **P < 0.01; ***P < 0.001; ****P < 0.0001.

The intestinal epithelium serves as one of the first lines of defense against CDI, as *C. difficile* toxins damage epithelial cells, leading to a loss of barrier integrity, release of proinflammatory cytokines, and immune cell infiltration (8). Numerous studies have shown that PGs fortify the intestinal epithelium and promote recovery after damage (22, 23, 25). Given our previous finding that misoprostol treatment reduces intestinal barrier permeability during CDI (18) and our data showing reduced epithelial damage (Fig. 1F), we examined whether protection by misoprostol is mediated by effects on the epithelium. Lipocalin-2 is a metal sequestering protein that is produced by epithelial cells and serves as a marker of epithelial inflammation (31). Given its role in epithelial responses, we measured the levels of lipocalin-2 at 2 days post-infection. We observed no significant differences in lipocalin-2 levels during CDI following misoprostol treatment (Fig. S2A). We next measured canonical markers of inflammation in isolated intestinal epithelial cells (IECs) at 2 days post-infection (32). Interestingly, we found no significant differences in expression of *Tnfa* or the monocyte- and neutrophil-recruiting chemokines *Ccl2* and *Cxcl1* (33, 34). While *Ccl2* expression was unchanged in isolated IECs, it was reduced in bulk colon tissue (Fig. 2F), suggesting that *Ccl2* expression during CDI is largely driven by non-epithelial cell populations that are modulated by misoprostol. However, we did observe decreased IEC expression of *Cxcl5*, a key neutrophil chemokine at mucosal sites (35) (Fig. S2B).

Based on the multifaceted roles that PGs play in shaping IEC responses, we examined whether misoprostol treatment during CDI impacts any of these key pathways (23, 25–27). One potential mechanism of protection is that misoprostol promotes expression of *Muc2*, a key mucin produced by epithelial cells that fortifies the physical barrier between IECs and the intestinal microbiota and is decreased in CDI patients (36). To test whether misoprostol impacts *Muc2* during CDI, we quantified expression in IECs at 2 days post-infection. Misoprostol treatment did not alter *Muc2* expression (Fig. S2C), suggesting that protection is not mediated through enhanced mucus production. Another potential mechanism of protection is that misoprostol promotes IEC proliferation during CDI, which can fortify the intestinal barrier. To test this, we quantified Ki67-positive IECs within the intestinal crypts in stained cecal sections at 2 days post-infection, as Ki67 is an established proliferation marker (37). Consistent with prior PG studies (25), misoprostol-treated uninfected mice (Miso) showed increased Ki67 staining compared to Abx mice. However, we did not observe any differences in epithelial proliferation between Cd and Cd + miso mice (Fig. S2D and E). Collectively, these data support a therapeutic role for misoprostol and indicate that its protective effects during CDI are not mediated through changes in *C. difficile* virulence, mucus production, or IEC proliferation. Instead, these findings suggest that misoprostol confers protection by modulating the host immune response to infection.

### Misoprostol reduces colonic monocyte and neutrophil infiltration during CDI

Our data demonstrate that misoprostol is anti-inflammatory during acute CDI, decreasing immune cell infiltration and levels of the neutrophil-derived protein calprotectin (Fig. 1F and H). Neutrophils can exacerbate tissue damage during CDI (10, 14); however, they are also critical in controlling disease as depletion of these cells increases CDI mortality (9). While the impacts of PGs on immune cell infiltration during CDI are unknown, PGE_2_ has been shown to reduce neutrophil infiltration into the lung during LPS-induced inflammation (38). Given these data and the important role of innate immune cell recruitment during infection, we postulated that misoprostol may modulate immune cell infiltration during CDI. To test this, we immunoprofiled innate immune populations in the colonic lamina propria (cLP) at 2 days post-infection (21) (Fig. S5A). We found that misoprostol treatment significantly reduced the total numbers of monocytes and neutrophils in the cLP (Fig. 2A through C). PGE_2_ has previously been shown to suppress neutrophil activation and TNF-α production during *T. gondii* infection (21). To assess whether misoprostol similarly alters neutrophil activation during CDI, we quantified TNF-α^+^ neutrophils in the cLP at 2 days post-infection (Fig. 2D). Although Cd + miso mice exhibited a higher proportion of TNF-α^+^ neutrophils compared to Cd mice, the total number of TNF-α^+^ neutrophils was reduced. Furthermore, TNF-α MFI in neutrophils did not differ between groups (Fig. 2E). Together, these findings suggest that misoprostol does not markedly alter neutrophil TNF-α expression on a per-cell basis, but instead reduces total neutrophil numbers in the cLP.

Next, to determine if misoprostol treatment affected expression of chemokines important for monocyte and neutrophil recruitment (33, 34), we quantified chemokine gene expression in colonic tissues. We observed significant reductions in the expression of *Ccl2* and *Cxcl5* in Cd + miso mice (Fig. 2F and G). Expression of *Cxcl1* and *Cxcl2*, which act primarily to recruit neutrophils, was unchanged with misoprostol treatment. Given the established roles of CCL2 and CXCL5 in monocyte and neutrophil recruitment (33, 35), these data suggest that misoprostol dampens colonic inflammation by limiting chemokine-driven recruitment of these innate immune cells to the cLP.

### Misoprostol alters systemic neutrophil levels during infection by reducing mobilization from the bone marrow

**Fig 3:**
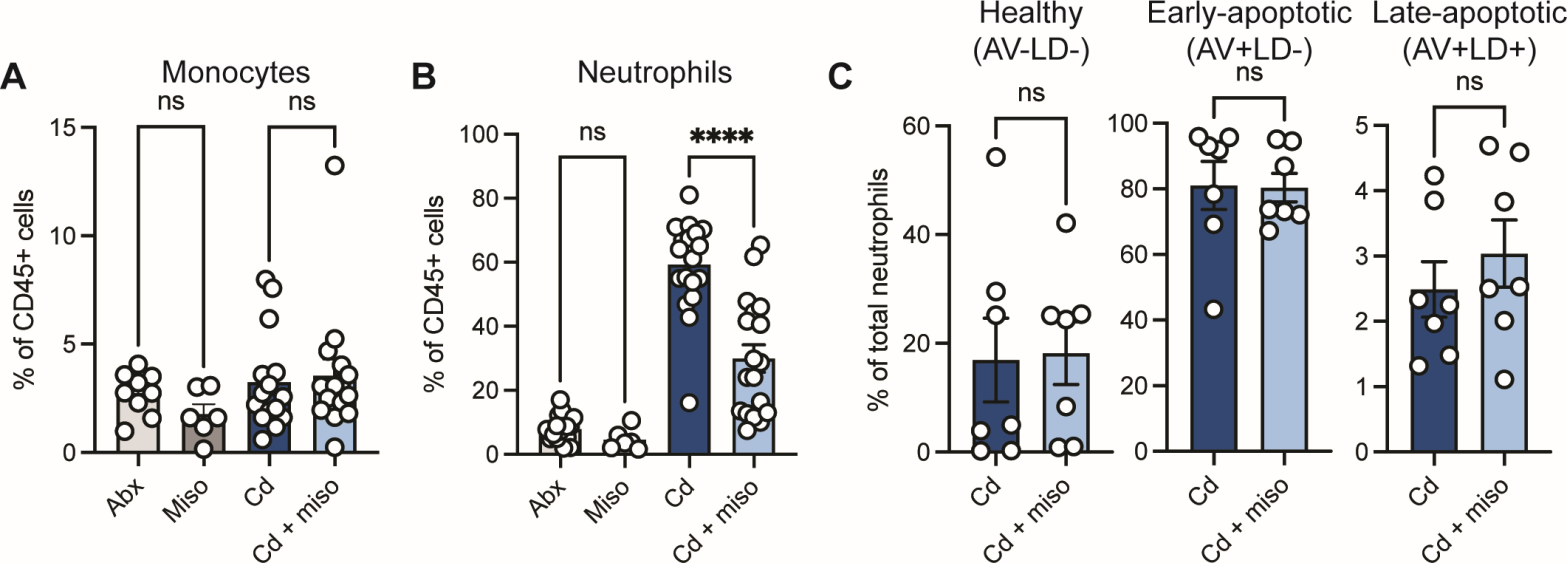
Misoprostol reduces systemic neutrophil levels during CDI. (**A–C**) Mice were infected with *C. difficile* (Cd) and treated with or without misoprostol (miso). Samples were collected 2 days post-infection. (**A and B**) Frequency of (**A**) monocytes and (**B**) neutrophils in the peripheral blood (*N* = 6–19 per group, representative of four independent experiments). (**C**) Annexin V (AV) and amine reactive dye (LD) staining in blood neutrophils. Percent of AV^−^LD^−^ (healthy), AV^+^LD^−^ (early-apoptotic), and AV^+^LD^+^ (late-apoptotic) neutrophils (*N* = 7 per group, representative of two independent experiments). (**A–C**) Each dot represents an individual mouse. Data are represented as mean ± SEM. Statistics by one-way ANOVA test with Tukey’s multiple comparisons (**A and B**) and Mann-Whitney *U* test (**C**). ns (not significant), *P* > 0.05; ****P < 0.0001.

Our data demonstrate that misoprostol alters recruitment of critical innate immune cells to the gastrointestinal tract during infection. To begin to define the underlying mechanisms of misoprostol-mediated immunomodulation during infection, we asked if the effects of misoprostol treatment were restricted to the gastrointestinal tract. Using flow cytometry, we quantified monocyte and neutrophil frequencies in the blood 2 days post-infection (39) (Fig. S5B). We did not find any significant differences in the frequencies of circulating monocytes between the groups (Fig. 3A), supporting that misoprostol reduces colonic infiltration of monocytes through altered chemokine expression. However, we observed marked reductions in the frequencies of circulating neutrophils in the blood of Cd + miso mice (Fig. 3B), suggesting that the effect of misoprostol on the innate immune response extends beyond the site of infection.

To determine how misoprostol reduces circulating neutrophil levels during infection, we next explored whether misoprostol impacts neutrophil survival or development. Neutrophils are short-lived cells primarily due to their ability to rapidly turn on apoptotic pathways that aim to balance tissue inflammation and homeostasis (40). To test the potential effects of misoprostol on neutrophil survival, we used annexin V (AV) and amine-reactive dye (LD) staining to discriminate healthy (AV^−^LD^−^), early-apoptotic (AV^+^LD^−^), and late-apoptotic (AV^+^LD^+^) neutrophils (41). We found no differences in viability or apoptotic states in blood neutrophils across treatment groups (Fig. 3C), suggesting that reduced circulating neutrophils is not due to altered survival.

**Fig 4:**
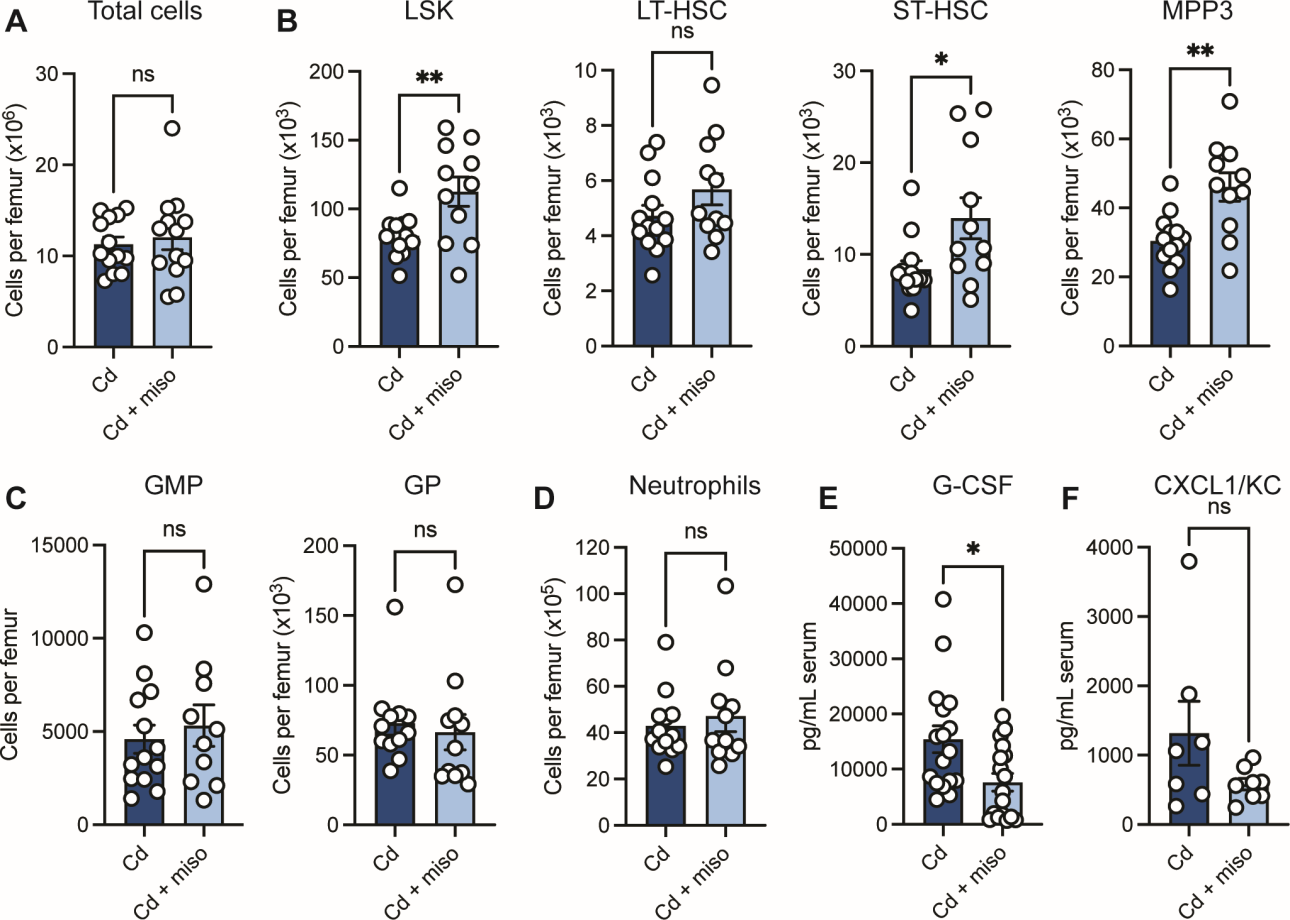
Misoprostol expands early hematopoietic progenitors without altering neutrophil development in the bone marrow. (**A–F**) Mice were infected with *C. difficile* (Cd) and treated with or without misoprostol (miso). Samples were collected 2 days post-infection. (**A**) Cellularity of femoral bone marrow (*N* = 13 per group). (**B**) LSK (Lin^−^Sca1^+^c-Kit^+^) cells per femur. These cells were further characterized as long-term HSCs (LT-HSC) (CD135^−^CD48^−^CD150^+^LSK), short-term HSCs (ST-HSC) (CD135^−^CD48^−^CD150^−^LSK), or multipotent progenitor 3 cells (MPP3) (CD135^−^CD48^+^CD150^−^LSK) (*N* = 10–13 per group). (**C**) Granulocyte-monocyte progenitor (GMP) (Lin^−^Ly6G^+^c-Kit^+^CD34^+^CD16/32^hi^Ly6C^−^CD135^−^CD115^−^) and granulocyte progenitor (GP) (Lin^−^Ly6G^+^c-Kit^+^CD34^+^CD16/32^hi^Ly6C^+^CD135^−^CD115^−^) cells per femur (*N* = 10–13 per group). (**D**) Neutrophils (Lin^+^Ly6G^+^Ly6C^+^) per femur (*N* = 11–13 per group). (**E**) Serum G-CSF levels quantified by ELISA (*N* = 17 per group). (**F**) Serum CXCL1/KC levels in mice measured by ELISA (*N* = 7–8 per group). (**A–F**) Each dot represents an individual mouse. Data are represented as mean ± SEM. Statistics by unpaired *t*-test (**A–E**) or Mann-Whitney *U* test (**F**). ns (not significant), *P* > 0.05; *P < 0.05; **P < 0.01.

Given these findings, we next investigated whether misoprostol impacts neutrophil development and expansion in the bone marrow. Neutrophils arise from self-renewing hematopoietic stem cells (HSCs), which give rise to all blood cell lineages (42). HSCs are defined as Lin^−^Sca1^+^c-Kit^+^ (LSK) cells and can differentiate into multipotent progenitors (MPPs), which are subdivided into distinct lineages. Among these, MPP3s are biased toward monocyte/granulocyte differentiation and give rise to granulocyte-monocyte progenitors (GMPs) and granulocyte progenitors (GPs), which can ultimately generate neutrophils (43). Therefore, to examine whether misoprostol impacts granulopoiesis during CDI, we analyzed these bone marrow progenitor populations at 2 days post-infection (44) (Fig. S6; Table S1). During CDI, total cellularity of the femoral bone marrow was unchanged between Cd and Cd + miso mice (Fig. 4A). However, Cd + miso mice exhibited a significant increase in total LSK cells and concurrent expansion of short-term HSCs and MPP3s (Fig. 4B), consistent with previous studies showing that PGE_2_ promotes HSC expansion (45–47). However, total numbers of downstream GMPs and GPs were unaffected (Fig. 4C), and there was no significant difference in total neutrophil numbers in the bone marrow between groups (Fig. 4D). These data suggest that, while misoprostol influences early hematopoietic progenitors during CDI, the reduction in circulating neutrophils is likely not due to decreased production but rather to altered egress from the bone marrow.

Based on these observations, we hypothesized that misoprostol may impair mobilization of bone marrow neutrophils into circulation. During fungal infection, increased PD-L1 expression on bone marrow neutrophils has been shown to restrict their release into the bloodstream (48). To determine whether a similar mechanism occurs during CDI with misoprostol treatment, we quantified PD-L1 expression on bone marrow neutrophils. Cd + miso mice exhibited a modest but significant increase in the frequency of PD-L1^+^ neutrophils compared to Cd mice (Fig. S3A). However, PD-L1 expression was detected on only a small fraction of the neutrophils, indicating that altered PD-L1 expression is unlikely the primary mechanism driving the reduction of circulating neutrophils.

Granulocyte colony-stimulating factor (G-CSF) is a critical regulator of granulopoiesis during both homeostasis and emergency granulopoiesis in response to infection (49). Along with its role in neutrophil development, G-CSF is also a potent inducer of neutrophil mobilization and release from the bone marrow into the bloodstream (28, 50, 51). During infection, G-CSF can be produced by multiple cell types, including intestinal epithelial cells, fibroblasts, macrophages, and endothelial cells, in response to inflammatory signals (52, 53). Additionally, chemokines, such as CXCL1/KC, are critical for neutrophil recruitment from the bone marrow into circulation (35). To test whether misoprostol impacts this process, we measured circulating levels of serum G-CSF at 2 days post-infection by ELISA. We found that Cd + miso mice had significantly reduced serum G-CSF levels compared to Cd mice (Fig. 4E). Notably, we did not observe any differences in serum CXCL1/KC levels between groups (Fig. 4F). Previous studies have shown that mice with impaired G-CSF signaling exhibit decreased circulating neutrophils despite normal levels of mature neutrophils in the bone marrow (28). Collectively, these data suggest that, during CDI, misoprostol may limit neutrophil mobilization from the bone marrow by dampening G-CSF production, resulting in decreased circulating neutrophils.

### Pharmacological modulation of neutrophil infiltration tunes disease severity during CDI

**Fig 5:**
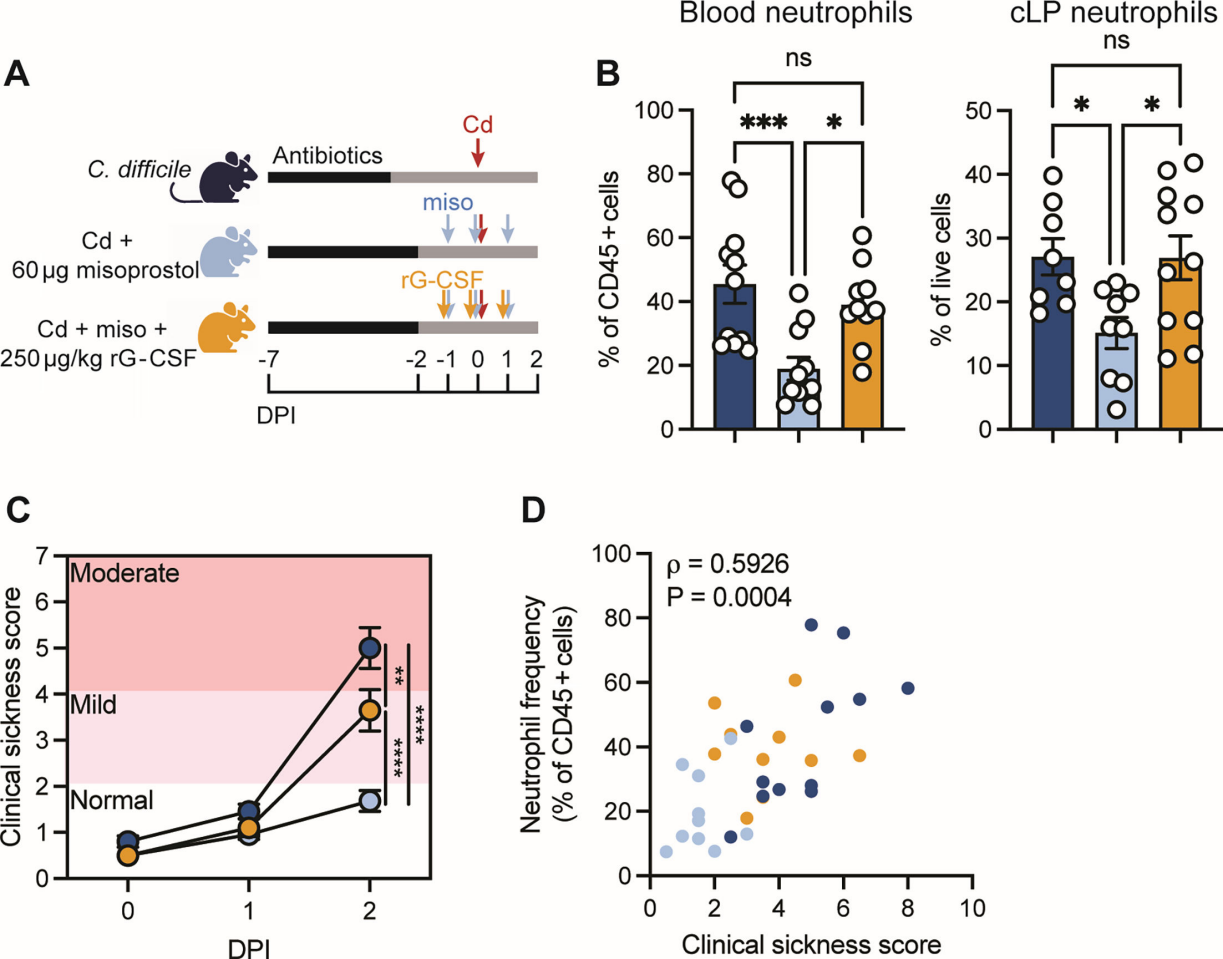
Pharmacological neutrophil modulation tunes CDI severity. (**A**) Schematic of experimental design. Mice were treated with antibiotics in their drinking water and infected with 1 × 10^5^ *C. difficile* spores (Cd). Misoprostol-treated mice were administered 60 μg misoprostol via intraperitoneal (i.p.) injection daily from −1 days post-infection (DPI) to 1 DPI. rG-CSF-treated mice received 250 μg/kg mouse recombinant G-CSF via i.p. injection daily concurrently with misoprostol. (**B**) Frequency of neutrophils in the peripheral blood and cLP at 2 DPI (*N* = 8–11 per group). (**C**) Composite score of weight loss, stool consistency, and behavior (*N* = 10–11 per group). (**D**) Two-sided Spearman correlation between blood neutrophil frequency and clinical sickness score of *C. difficile-*infected mice at 2 DPI. Each dot represents an individual mouse and is color-coded by treatment group (Spearman *r* = 0.5926, *N* = 32). (**B and C**) Data are represented as mean ± SEM and representative of two independent experiments. Statistics by one-way (**B**) or two-way (**C**) ANOVA with Tukey’s multiple comparisons. ns (not significant), *P* > 0.05; *P < 0.05; **P < 0.01; ***P < 0.001; ****P < 0.0001.

Our data suggest that misoprostol reduces CDI disease severity by reducing circulating neutrophils and recruitment to the site of infection through modulation of G-CSF levels and chemokine expression. Because G-CSF promotes neutrophil mobilization into circulation (28, 50, 51), we hypothesized that G-CSF supplementation would restore blood neutrophils and counteract the effects of misoprostol. To test this, we treated mice with misoprostol and 250 μg/kg recombinant mouse G-CSF (rG-CSF) daily beginning one day prior to infection with *C. difficile* (Cd + miso + rG-CSF) (Fig. 5A). We observed that rG-CSF treatment restored neutrophil frequencies in the blood and cLP in Cd + miso + rG-CSF mice to those observed in Cd mice at 2 days post-infection (Fig. 5B). Additionally, rG-CSF treatment did not impact monocyte frequencies (Fig. S4A) or expression of monocyte and neutrophil chemokines in the colon (Fig. S4B and C). Notably, this treatment and concurrent increases in systemic and colonic neutrophil levels significantly increased severity of disease following infection. However, disease was not as severe as Cd mice (Fig. 5C), suggesting that misoprostol reduces CDI severity in part by limiting neutrophil infiltration but also exerts other protective effects that further mitigate disease.

Finally, to contextualize these data and assess the relationship between neutrophils and *C. difficile*-associated disease, we examined correlations between circulating neutrophil frequencies in the blood and CDI severity. Across infected mice, circulating neutrophils positively correlated with disease severity. Strikingly, reduced neutrophil frequencies were associated with decreased severity in Cd + miso mice. Additionally, misoprostol and rG-CSF treatment restored both neutrophil levels and severity to intermediate levels between Cd + miso and Cd mice (Fig. 5D). Together, these findings underscore the pivotal role of neutrophil abundance in driving CDI pathology and highlight the potential of modulating innate immune responses as a treatment strategy to combat CDI.

## DISCUSSION

In this study, we identify a novel mechanism by which the innate immune system can be modulated to improve CDI outcomes. CDI remains difficult to treat, with high recurrence rates and increasing antibiotic resistance. Although antibiotics are used to treat infections, they perpetuate microbiota disruption and do not address the host inflammatory responses that strongly correlate with disease severity (4, 5). We previously reported that misoprostol improves CDI survival in mice, but the mechanism of protection remained unclear (18). Here, we demonstrate that misoprostol reduces acute CDI severity by limiting G-CSF production, thereby reducing neutrophil availability and subsequent infiltration into the colon. Despite these protective effects during acute CDI, misoprostol did not reduce CDI recurrence in our model (Fig. S1), highlighting the context dependence of immunomodulation and suggesting that additional ecological factors, including restoration of colonization resistance, are likely to play important roles in shaping recovery and susceptibility to relapse.

PGs have key roles in intestinal homeostasis, intestinal wound repair, and mucosal protection, but their function in CDI has not been extensively explored (16). We found that misoprostol reduces epithelial injury during CDI (Fig. 1F). However, we did not detect changes in epithelial inflammatory or mucus-associated gene expression or IEC proliferation at 2 days post-infection (Fig. S2). Notably, misoprostol did increase IEC proliferation in uninfected mice (Fig. S2E), suggesting that earlier or transient effects on the epithelium prior to CDI could contribute to barrier fortification upon infection. However, further work will be needed to further test this hypothesis.

Our data highlight immunomodulation as the dominant mechanism of misoprostol-mediated protection. E-series PGs, such as PGE_2_, have been shown to dampen innate immune cell activation and proinflammatory cytokine production during infection (17, 21, 38, 54). Consistent with this, misoprostol reduced colonic *Tnfa* expression and levels of the neutrophil-derived inflammatory marker calprotectin (Fig. 1G and H). Notably, misoprostol also reduced total neutrophils in the lamina propria (Fig. 2C) and neutrophil frequency in the blood (Fig. 3B), which were previously unreported. Misoprostol also decreased expression of chemokines that drive neutrophil recruitment (Fig. 2G). Since chemokine gradients orchestrate neutrophil trafficking, suppressing these signals may help limit intestinal infiltration. Neutrophils are a hallmark of CDI and correlate with greater disease severity (5), yet the mechanisms linking neutrophil influx to tissue damage during CDI remain incompletely defined. Additionally, excessive recruitment of neutrophils to the colonic mucosa can cause crypt abscesses and transepithelial migration, leading to increased intestinal barrier permeability (55, 56). Hence, a reduction in neutrophil infiltration and potentially activation likely contributes to the decrease in colonic inflammation and the reduced epithelial tissue damage we observe.

PGE_2_ has a well-established role in hematopoiesis, promoting stem cell proliferation and survival (46, 47). Here, we found that misoprostol, a PGE_2_ mimetic, phenocopies these effects, increasing the total number of HSCs and reshaping the early immune progenitor pool, expanding ST-HSCs and MPP3s as seen with PGE_2_. Surprisingly, misoprostol treatment did not alter total numbers of later neutrophil progenitors or bone marrow neutrophils (Fig. 4A through D). This suggests that misoprostol’s impact on granulopoiesis is unlikely to directly account for the reduced neutrophils observed in the blood and colon and instead points to altered mobilization.

G-CSF is a key cytokine that drives neutrophil development and mobilization during emergency granulopoiesis (28, 51). Here, we show that Cd + miso mice had reduced circulating G-CSF (Fig. 4E), potentially limiting neutrophil mobilization from the bone marrow. Administration of recombinant G-CSF restored blood neutrophil frequencies and, unexpectedly, also enhanced neutrophil infiltration into the colon (Fig. 5B). Despite these increases, disease severity of Cd + miso + rG-CSF mice only reached an intermediate level between Cd + miso and Cd mice (Fig. 5C), highlighting that reduced neutrophil mobilization is a major, but not exclusive, contributor to misoprostol-mediated protection and suggesting that misoprostol’s protective effects extend beyond neutrophil abundance alone and reflect broader immunomodulatory actions. Strikingly, we observed a positive correlation between higher circulating neutrophil frequencies and increased CDI severity (Fig. 5D), supporting the concept that excessive neutrophil recruitment exacerbates pathology. Additional studies will be necessary to further understand how misoprostol impacts G-CSF production and subsequent effects on neutrophil mobilization. Although multiple cell types, including IECs, fibroblasts, and immune cells, such as monocytes and neutrophils, are capable of producing G-CSF (52, 57), the major source of G-CSF during acute CDI and the mechanism by which misoprostol reduces its production remain unknown. One possibility is that misoprostol indirectly limits G-CSF production by reducing expansion or recruitment of G-CSF-producing immune cells, establishing a feedback loop in which reduced G-CSF further restricts neutrophil mobilization from the bone marrow. Alternatively, misoprostol may directly modulate G-CSF production through EP receptor signaling in G-CSF-producing cells (57, 58), or indirectly by altering upstream inflammatory pathways that regulate G-CSF expression, including IL-17-dependent signaling (59).

Collectively, this work identifies a novel mechanism by which misoprostol protects against CDI and provides a new framework for understanding how neutrophils contribute to CDI severity. By demonstrating that modulation of neutrophil mobilization and infiltration can markedly alter CDI outcomes, this study emphasizes the balance of neutrophil biology during CDI and establishes this innate immune cell type as a potential therapeutic target for host-directed therapy. In this study, misoprostol was administered prior to and throughout acute infection, defining its effects in a prophylactic rather than therapeutic context. Consistent with this, misoprostol did not reduce recurrence when given during acute infection, highlighting the context dependence of immunomodulatory strategies and the importance of treatment timing. Because misoprostol primarily reduces host inflammatory responses rather than promoting pathogen clearance, its impact on recurrence may differ when administered during the recovery phase. Additionally, the dose and administration used here were selected to define mechanism rather than to model clinically equivalent exposure. In this study, misoprostol was administered via i.p. injection to ensure consistent and systemic exposure during acute infection; however, in clinical settings, it is administered orally. Notably, oral misoprostol has been evaluated in a recent clinical trial as a strategy to reduce CDI recurrence (60). Although this study was limited by enrollment challenges, our mechanistic findings provide renewed support for continued investigation of misoprostol, or other neutrophil-modifying treatments, in clinical settings. Together, these findings highlight neutrophil infiltration as a critical driver of CDI pathology and establish misoprostol as a promising host-directed therapeutic strategy. While additional studies are needed to define its effects in humans, this work provides a foundation for developing targeted interventions that complement current treatments for CDI.

## MATERIALS AND METHODS

### Animal and experimental models of *C. difficile* infection

C57BL/6 male mice were infected with *C. difficile* as previously described (7). Mice were administered 1 × 10^5^ spores of *C. difficile* CD196 via oral gavage. Mice were monitored daily for survival, weight loss, and clinical sickness. Clinical sickness was quantified as previously described, consisting of a composite score of weight loss, stool consistency, and moribund behavior, including responsiveness and ability to ambulate (29).

CDI relapse was induced as previously described (61). Mice were assessed daily for CDI relapse following vancomycin cessation. Relapse was defined as having detectable toxin in stool. Mice were monitored daily and euthanized when they appeared moribund or when weight loss exceeded 20% of their starting weight.

### Misoprostol and rG-CSF treatment

Misoprostol was purchased from Cayman Chemicals (Ann Arbor, MI) in methyl acetate. The solvent was evaporated under nitrogen, and the misoprostol was resuspended in PBS immediately before each administration. Sixty micrograms of misoprostol was administered daily via intraperitoneal (i.p.) injection starting one day prior to infection. This dose was selected based on our prior study demonstrating a maximal survival benefit during CDI at this dose (18).

Mouse G-CSF recombinant protein (rG-CSF) (PeproTech) was resuspended in water according to manufacturer’s instructions. rG-CSF at 250 μg/kg was administered via i.p. injection daily starting one day prior to infection. This dose was selected based on prior studies demonstrating robust neutrophil mobilization *in vivo* (28, 62).

### *C. difficile* burdens and toxin titers from feces

To quantify *C. difficile* burdens, fecal samples were weighed, homogenized in sterile PBS, and plated on taurocholate cycloserine cefoxitin fructose agar (TCCFA) under anaerobic conditions. *C. difficile* toxin titers were quantified using a Vero cell rounding assay as previously described (63). Toxin titers were calculated as the reciprocal value of the highest dilution with 100% cell rounding. Both *C. difficile* CFU and toxin titers were normalized to stool weight.

### Histological analysis

Samples were fixed in 10% formalin and embedded in paraffin as previously described (7). Slides were stained with hematoxylin and eosin and assigned a disease score by a blinded pathologist based on previously described criteria (64). Histological scores are reported as a cumulative score of three independent criteria: epithelial damage, inflammation, and edema.

Ki67 staining was performed as previously described (65). The immunohistochemistry slides were scanned at 40×. Using QuPath 5.1, an object classifier was built to distinguish epithelial cells from immune cells and other cells using a random forest classifier after numerous annotations from an image created from samples of 15 case images. The percentage of Ki67-positive epithelial cells was determined by deploying the positive cell detection and built object classifier tools (66).

### Fecal and serum ELISAs

Lipocalin-2 and calprotectin were quantified from stool using mouse lipocalin-2/NGAL and S100A8/S100A9 R&D DuoSet ELISA kits according to manufacturer’s instructions. Fecal pellets were homogenized in 1 mL sterile PBS and centrifuged at 4,000 rpm for 5 min. Supernatants from *C. difficile* were diluted 1:100 for lipocalin-2 and 1:50 for calprotectin quantification. Protein concentrations were normalized to grams of feces.

CXCL1/KC and G-CSF were quantified from serum using R&D DuoSet ELISA kits, according to the manufacturer’s instructions. Serum was collected and diluted 1:10 for CXCL1/KC and 1:30 or 1:50 for G-CSF quantification.

### RNA extraction and RT-qPCR

RNA was extracted using RNeasy Mini Kit (QIAgen), according to the manufacturer’s instructions. cDNA was synthesized using M-MLV reverse transcriptase (Promega), according to the manufacturer’s instructions. RT-qPCR was performed using iQ SYBR reagents (Bio-Rad), and data were normalized to the expression of *Gapdh*. Primers are listed in Table S3.

### Isolation of cells for flow cytometry

Intestinal epithelial cells (IECs) and colonic lamina propria cells (cLPs) were isolated from mice as previously described (67). Briefly, colons and ceca were collected, opened longitudinally, washed in PBS, and cut into 1 cm pieces. The tissue was incubated in HBSS with 2 mM EDTA and shaken for 10 min at 150 rpm to dissociate IECs. Supernatants containing IECs were filtered through 70 μm cell strainer and used for RT-qPCR. The remaining tissue pieces were washed, minced, and incubated in digestion buffer for 45 min at 37°C. Cell suspensions were filtered through 70 μm cell strainer and stained directly.

Blood was obtained via cardiac puncture and collected in heparin-containing tubes. Following red blood cell lysis with ACK lysis buffer, cells were washed and pelleted at 400 × *g* for 5 min. ACK lysis was repeated until cell pellets were no longer red. Cells were enumerated and transferred to U-bottom 96-well plate for staining.

Both femurs were collected from mice for bone marrow isolation as previously described (68). Cells were enumerated and equivalent numbers of cells were transferred into a U-bottom 96-well plate for staining.

### Flow cytometry and antibodies

Cell suspensions were prepared as described above and stained with LIVE/DEAD Fixable Aqua (1:600) or Fixable Viability Dye 660 (1:1,000) in PBS. Cells were then suspended in Fc block (5 µg/mL CD16/32 in FACS buffer). If CD16/32 was stained for, the staining antibody was substituted for the unconjugated antibody. Antibodies used are listed in Table S2, and gating strategies are described in Fig. S5 and S6. To assess neutrophil viability, cells were stained with antibodies against annexin V according to the manufacturer’s instructions. Early-apoptotic neutrophils were defined as annexin V^+^ Fixable Viability Dye 660^−^, and late-apoptotic neutrophils were defined as annexin V^+^ Fixable Viability Dye 660^+^. Cells were stained in FACS buffer for all samples. Samples were run on a Cytek Aurora, and data were analyzed using FlowJo software.

### Statistical analysis

Statistical analyses were performed using GraphPad Prism version 10.6.0. Specific tests are reported in the figure legends.

## Data Availability

All data needed to evaluate the conclusions in the paper are available in the main text or the supplemental materials.

## SUPPLEMENTAL MATERIAL

Supplemental material mbio.03430-25-s0001.docx Figures S1 to S6; Tables S1 to S3.

ASM does not own the copyrights to Supplemental Material that may be linked to, or accessed through, an article. The authors have granted ASM a non-exclusive, world-wide license to publish the Supplemental Material files. Please contact the corresponding author directly for reuse.
