# Supplementary material for: Pharmacological reduction of neutrophil infiltration reduces Clostridioides difficile infection severity
Source: mBio. 2026 Mar 30;17(5):e03430-25. doi: 10.1128/mbio.03430-25 (PMC13170173; doi:10.1128/mbio.03430-25)
Supplement: Supplemental material — Figures S1 to S6; Tables S1 to S3. [file mbio.03430-25-s0001.docx]

Supplementary Materials for

**Pharmacological reduction of neutrophil infiltration reduces *Clostridioides difficile* infection severity**

Orlaith Keenan^1,2^, Joshua Soto Ocaña^1,2^, Alexa Semon^1,2,3^, Tiffany H. Zhou^1,2^, Kassy Donohoe^2^, Emma E. Furth^1^, Gavyn Chern Wei Bee^4^, Daniel L. Aldridge^3,5^, Juliana Diamantino^2^, Christopher A. Hunter^3,5^, Ken Cadwell^3,4,5^, David M. Aronoff^6^, Joseph P. Zackular^1,2,3,7^*

Corresponding authors:

Joseph P. Zackular: joseph.zackular@pennmedicine.upenn.edu


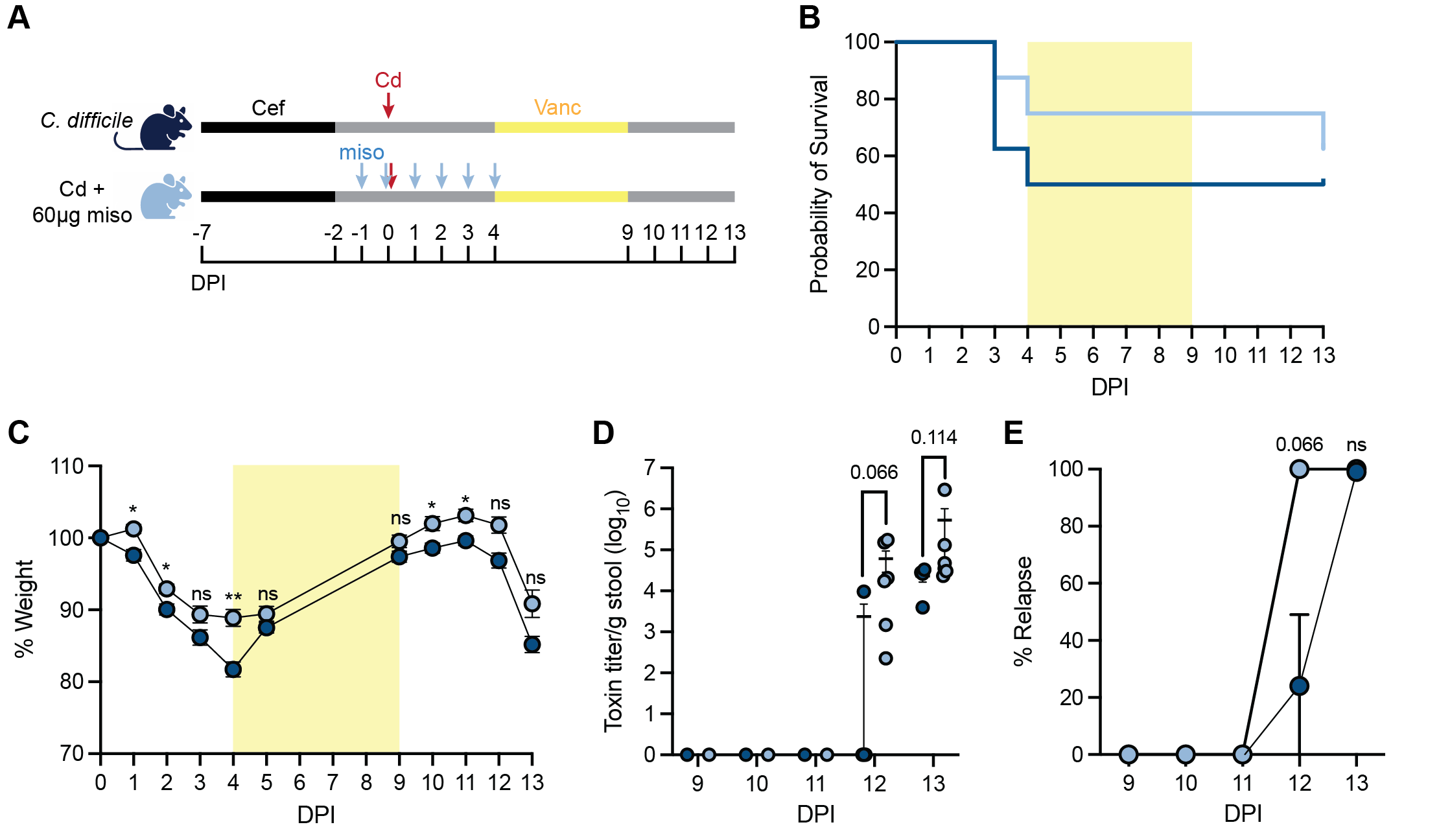
**Fig. S1. Misoprostol does not reduce susceptibility to CDI relapse.** (**A**) Experimental schematic. Mice were treated with cefoperazone (cef) for 5 days and infected with *C. difficile* (Cd) two days later. Mice treated with misoprostol (Cd + miso) were administered 60µg misoprostol daily from -1 days post infection (DPI) to 4 DPI. Mice were then treated with vancomycin (vanc) from 4 DPI to 9 DPI and monitored for relapse following vancomycin cessation. (**B**) Probability of survival. Yellow box indicates vancomycin treatment. (**C**) Percent of starting weight throughout the infection. (**D**) Toxin titers in stool of mice following vancomycin cessation. (**E**) Percent of mice with relapsing infection after vancomycin cessation. Relapse was defined as detectable toxin in stool. (**C-D**) Data are represented as mean ± SEM and N = 8 mice per group. Statistics by multiple Mann-Whitney U tests with Holm-Sidak’s multiple comparisons. ns (not significant) P>0.05, *P<0.05, **P<0.01.


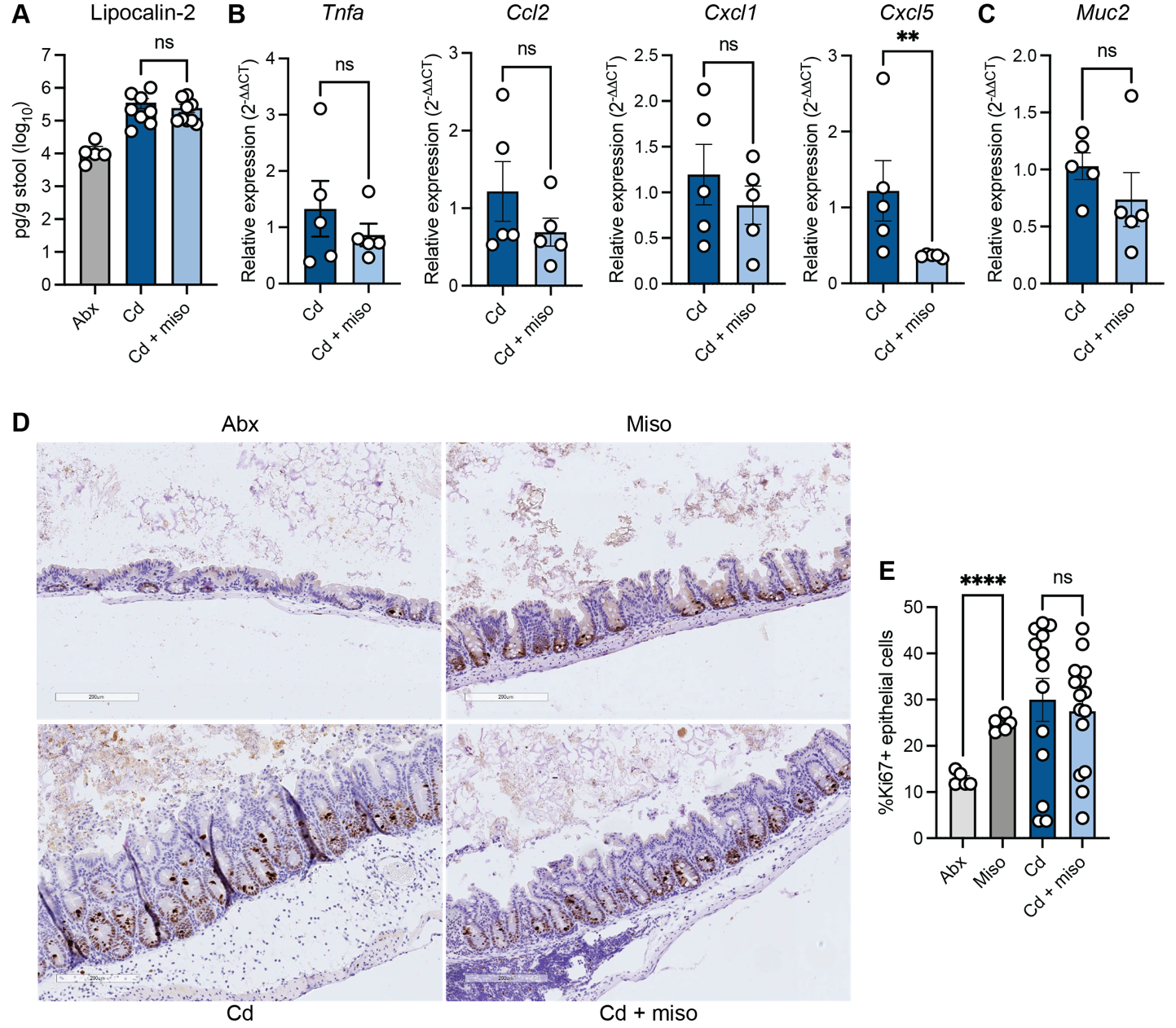


Fig. S2. Misoprostol-mediated protection is not associated with the intestinal epithelial response to infection. (A) Fecal lipocalin-2 levels of antibiotic-treated uninfected controls (Abx,), *C. difficile-*infected mice (Cd), and *C. difficile*-infected and misoprostol-treated mice (Cd + miso) at two days post-infection (DPI) measured by ELISA (N = 5 – 9 per group). (B and C) RT-qPCR of (B) proinflammatory cytokine and chemokine and (C) mucin-related gene expression in IECs harvested from mice at 2 DPI. Data relative to average *Gapdh* expression of Cd mice (N = 5 per group). (D) Representative images of Ki67 staining in ceca from antibiotic-treated (Abx), misoprostol-treated (Miso), *C. difficile* infected (Cd), and *C. difficile* infected and misoprostol-treated mice (Cd + miso). (E) Quantification of Ki67+ epithelial cells (N = 5 – 15 per group, representative of three independent experiments). (A–E) Data are represented as mean ± SEM. Statistics by one-way ANOVA test with Dunnett’s T3 multiple comparisons (A, E) and Mann-Whitney U test (B-C). ns (not significant) P>0.05, **P<0.01, ****p<0.0001.


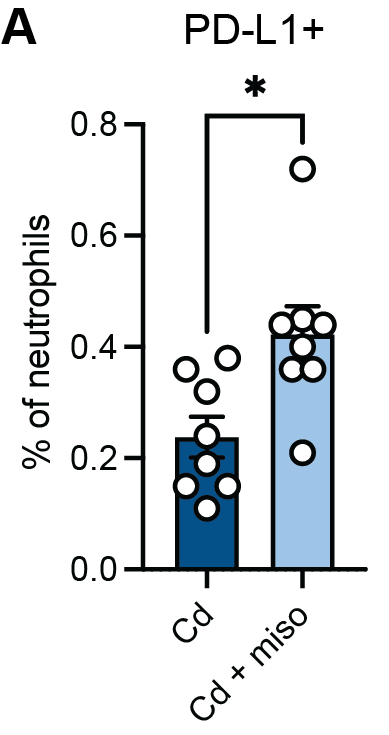


**Fig. S3. Misoprostol treatment increases PD-L1 expression on bone marrow neutrophils during CDI.** (**A**) Frequency of PD-L1+ neutrophils in the bone marrow (N = 8 per group). Each dot represents an individual mouse. Data are represented as mean ± SEM. Statistics by unpaired t test. *P<0.05.


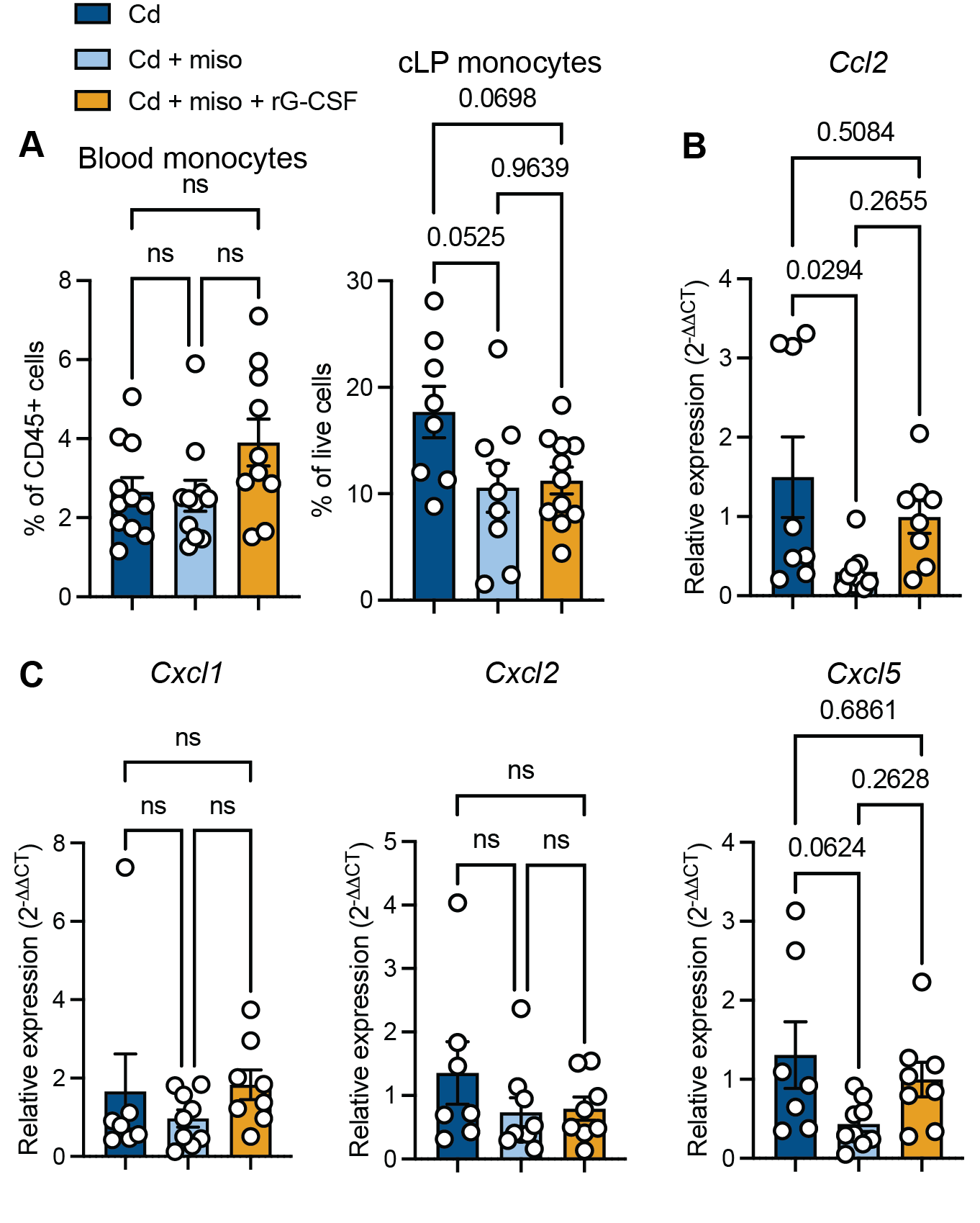


Fig. S4. rG-CSF treatment does not impact monocyte frequencies or colonic chemokine expression during CDI. (A) Frequency of monocytes in the peripheral blood and cLP at 2 DPI (N = 8 – 11 per group). (B and C) RT-qPCR of (B) monocyte- and (C) neutrophil-recruiting chemokines in bulk colon tissue harvested from mice. Data relative to average *Gapdh* expression of Cd mice (N = 6 – 9 per group, representative of three independent experiments). (A-C) Data are represented as mean ± SEM and representative of two independent experiments. Statistics by one-way ANOVA with Tukey’s multiple comparisons.


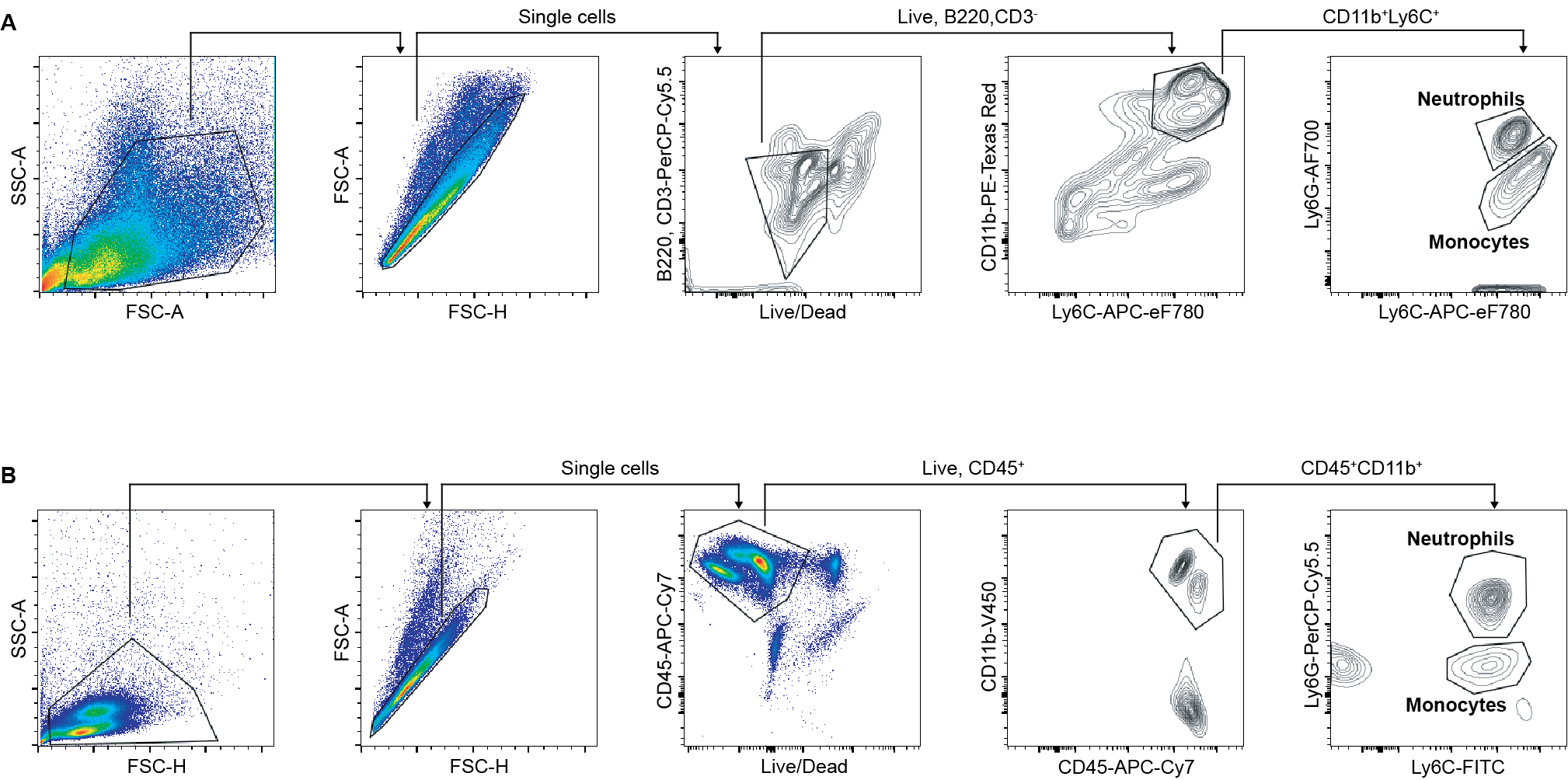


Fig. S5. Gating of neutrophils and monocytes in the cLP and blood. Representative flow plots from the (A) cLP or (B) blood are shown indicating the gating strategy used to identify neutrophils and monocytes.


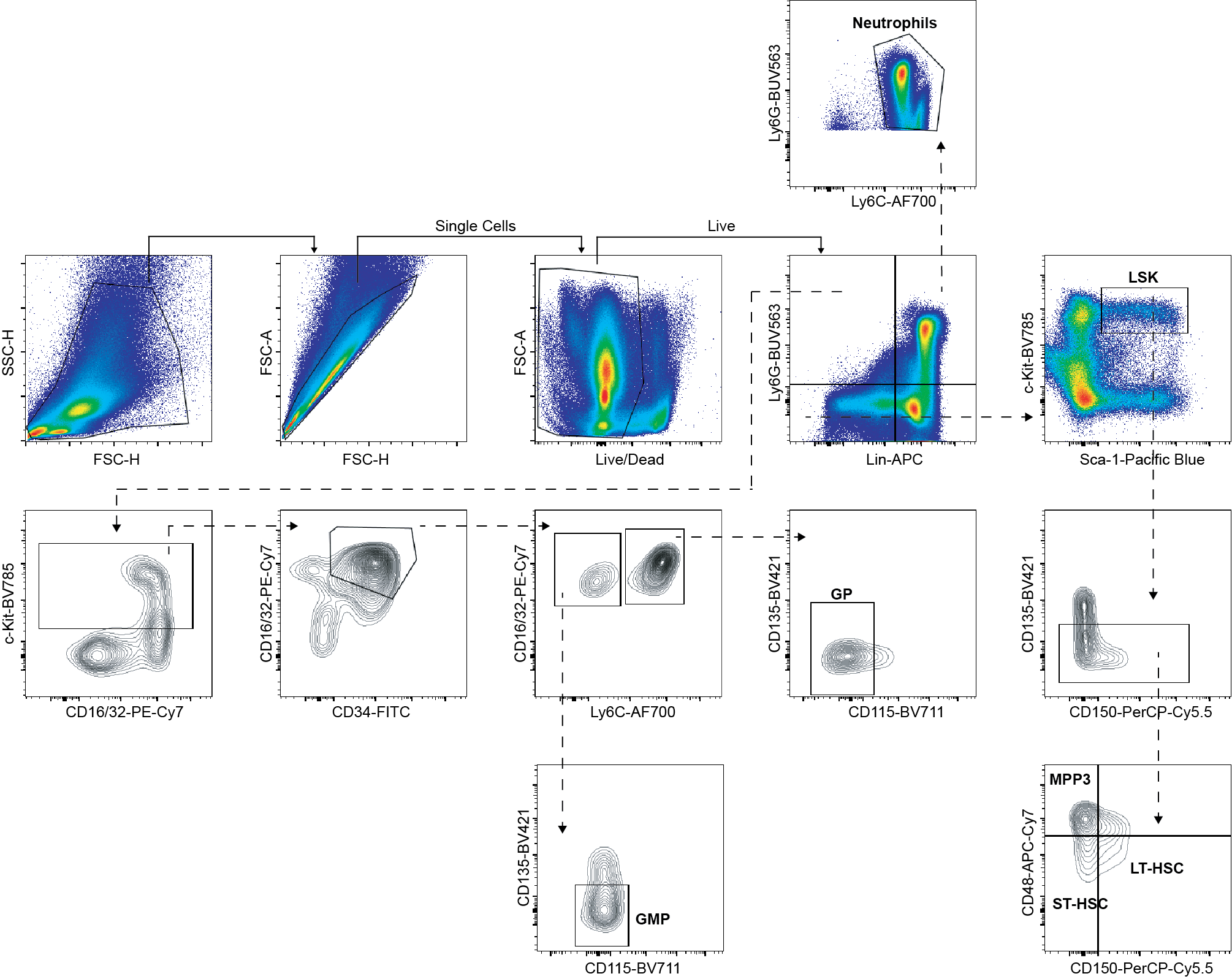


Fig. S6. Gating of HSCs and neutrophil progenitors in the bone marrow. Representative flow plots from the bone marrow are shown indicating the gating strategy used to identify various cell populations.

| **Cell type** | **Surface Markers** |
| --- | --- |
| LSK | Lin(CD3ε, CD11b, CD45R/B220, TER119)^-^, Ly6G^-^, Sca-1^+^, c-Kit^+^ |
| Long-term hematopoietic stem cell (LT-HSC) | LSK, CD135^-^, CD48^-^, CD150^+^ |
| Short-term hematopoietic stem cell (ST-HSC) | LSK, CD135^-^, CD48^-^, CD150^-^ |
| Multipotent progenitor 3 (MPP3) | LSK, CD135^-^, CD48^+^, CD150^-^ |
| Granulocyte-monocyte progenitor (GMP) | Lin(CD3ε, CD11b, CD45R/B220, TER119)^-^, Ly6G^+^, c-Kit^+^, CD16/32^hi^, CD34^+^, Ly6C^-^, CD135^-^, CD115^-^ |
| Granulocyte progenitor (GP) | Lin(CD3ε, CD11b, CD45R/B220, TER119)^-^, Ly6G^+^, c-Kit^+^, CD16/32^hi^, CD34^+^, Ly6C^+^, CD135^-^, CD115^-^ |
| Neutrophil | Lin(CD3ε, CD11b, CD45R/B220, TER119)^+^, Ly6G^+^, Ly6C^+^ |

Table S1. Surface markers of cells identified in the bone marrow.

| **Staining panel(s)** | **Marker** | **Fluorophore** | **Clone** | **Supplier** | **Dilution** |
| --- | --- | --- | --- | --- | --- |
| cLP, Blood, BM | Viability | LIVE/DEAD Fixable Aqua | - | Invitrogen | 1:600 |
| Blood | Viability | Fixable Viability Dye 660 | - | Invitrogen | 1:1000 |
| cLP | CD3 | PerCP-eF710 | 17A2 | Invitrogen | 1:200 |
| cLP | B220 | PerCP/Cy5.5 | RA3-6B2 | eBioscience | 1:200 |
| cLP | CD11b | PE-Texas Red | M1/70.15 | Invitrogen | 1:300 |
| cLP | Ly6G | AF700 | 1A8 | Biolegend | 1:300 |
| cLP | Ly6C | APC-eF780 | HK1.4 | eBioscience | 1:800 |
| cLP | TNFα | FITC | MP6-XT22 | eBioscience | 1:400 |
| Blood | CD45 | APC/Cy7 | 30-F11 | BD | 1:150 |
| Blood | CD11b | V450 | M1/70 | BD | 1:150 |
| Blood | Ly6G | PerCP/Cy5.5 | 1A8 | BD | 1:150 |
| Blood | Ly6C | FITC | HK1.4 | Biolegend | 1:150 |
| Blood | Annexin V | PE/Cy7 | - | Invitrogen | 1:20 |
| BM | CD16/32 | PE/Cy7 | 93 | Invitrogen | 1:200 |
| BM | CD3ε | APC | 145-2C11 | Biolegend | 1:200 |
| BM | CD11b | APC | M1/70 | Biolegend | 1:200 |
| BM | CD45R/B220 | APC | RA3-6B2 | Biolegend | 1:200 |
| BM | TER-119 | APC | TER-119 | Biolegend | 1:200 |
| BM | CD115 | BV711 | AFS98 | Biolegend | 1:200 |
| BM | Sca1 | Pacific Blue | E13-161.7 | Biolegend | 1:200 |
| BM | c-Kit | BV785 | 2B8 | Biolegend | 1:200 |
| BM | CD48 | APC/Cy7 | HM48-1 | Biolegend | 1:200 |
| BM | CD150 | PerCP/Cy5.5 | TC15-12F12.2 | Biolegend | 1:100 |
| BM | CD34 | FITC | RAM34 | eBioscience | 1:100 |
| BM | CD135 | BV421 | A2F10 | Biolegend | 1:100 |
| BM | Ly6C | AF700 | HK1.4 | Biolegend | 1:300 |
| BM | Ly6G | BUV563 | 1A8 | BD | 1:300 |
| BM | CD274 (PD-L1) | PE | B7-H1 | eBioscience | 1:100 |

**Table S2. Antibodies used in this study.** Antibodies and dilutions used in this study for staining cells in the colonic lamina propria (cLP), blood, and bone marrow (BM).

| **Gene** | **Forward primer sequence (5’-3’)** | **Reverse primer sequence (5’-3’)** |
| --- | --- | --- |
| *Ccl2* | GCTACAAGAGGATCACCAGCAG | GTCTGGACCCATTCCTTCTTGG |
| *Cxcl1* | TCCAGAGCTTGAAGGTGTTGCC | AACCAAGGGAGCTTCAGGGTCA |
| *Cxcl2* | CATCCAGAGCTTGAGTGTGACG | GGCTTCAGGGTCAAGGCAAACT |
| *Cxcl5* | CCGCTGGCATTTCTGTTGCTGT | CAGGGATCACCTCCAAATTAGCG |
| *Tnfa* | GCCTCTTCTCATTCCTGCTTG | CTGATGAGAGGGAGGCCATT |
| *Muc2* | AAACTGCTCTCTGGACTGCC | TTGGTTGGTGTGCTGAGTGT |
| *Gapdh* | AGCAAGGACACTGAGCAAGAG | GCAGCGAACTTTATTGATGGT |

**Table S3. Primers used in this study.**
